# Retraction: Rapid and selective detection of aluminum ion using 1,2,3-triazole-4,5-dicarboxylic acid-functionalized gold nanoparticle-based colorimetric sensor

**DOI:** 10.1039/d4ra90132k

**Published:** 2025-01-07

**Authors:** Shengliang Zhao, Liqiong Chen, Feiyan Liu, Yongyao Fan, Yiheng Liu, Yulai Han, Yunfei Hu, Jingyun Su, Chunyan Song

**Affiliations:** a College of New Materials and New Energies, Shenzhen Technology University Shenzhen Guangdong Province China chenliqiong@sztu.edu.cn; b College of Applied Technology, Shenzhen University Nanshan District Shenzhen Guangdong Province China; c Analysis and Testing Center, Shenzhen Technology University Pingshan District Shenzhen Guangdong Province China

## Abstract

Retraction of ‘Rapid and selective detection of aluminum ion using 1,2,3-triazole-4,5-dicarboxylic acid-functionalized gold nanoparticle-based colorimetric sensor’ by Shengliang Zhao *et al.*, *RSC Adv.*, 2021, **11**, 30635–30645, https://doi.org/10.1039/D1RA04834A.

The Royal Society of Chemistry, with the agreement of the authors, hereby wholly retracts this *RSC Advances* article due to errors in Fig. 3 and Scheme 1.

The TEM images in Fig. 3b and d have partial overlap. In Scheme 1 the vial for citrate-AuNPs and the vial for TADA-AuNPs are identical.

The authors state that these oversights were due to the shortcomings in data management in the laboratory and presentation in the manuscript, and they have requested retraction of this article.

The authors sincerely apologize for any confusion, inconvenience and negative impact to readers.

Given the significance of these concerns, the findings presented in this paper are no longer reliable.

Signed: Shengliang Zhao, Liqiong Chen, Feiyan Liu, Yongyao Fan, Yiheng Liu, Yulai Han, Yunfei Hu, Jingyun Su, Chunyan Song

12th December 2024

Retraction endorsed by Laura Fisher, Executive Editor, *RSC Advances*

